# Association of *DRD2, DRD4* and *COMT* genes variants and their gene-gene interactions with antipsychotic treatment response in patients with schizophrenia

**DOI:** 10.1186/s12888-023-05292-9

**Published:** 2023-10-25

**Authors:** Narges Taheri, Rokhshid Pirboveiri, Mehdi Sayyah, Mahdi Bijanzadeh, Pegah Ghandil

**Affiliations:** 1 Cellular and Molecular Research Center, Medical Basic Sciences Research Institute, Ahvaz Jundishapur University of Medical Sciences, Ahvaz, Iran; 2 Department of Medical Genetics, School of Medicine, Ahvaz Jundishapur University of Medical Sciences, Ahvaz, Iran; 3 Salamat hospital, Ahvaz Jundishapur University of Medical Sciences, Ahvaz, Iran; 4 Education Development Center (EDC), Ahvaz Jundishapur University of Medical Sciences, Ahvaz, Iran; 5 Thalassemia and Hemoglobinopathy Research Center, Health research Institute, Ahvaz Jundishapur University of Medical Sciences, Ahvaz, Iran; 6 Diabetes Research Center, Health Research Institute, Ahvaz Jundishapur University of Medical Sciences, Ahvaz, Iran

**Keywords:** Schizophrenia, Antipsychotic treatment, *DRD2*, *DRD4*, *COMT*, Gene, Polymorphism

## Abstract

Antipsychotic drugs are the first line of treatment in schizophrenia; although antipsychotic responses indicate a wide interindividual variety in patients with schizophrenia. This study aimed to investigate the association between four polymorphisms in *DRD2, DRD4* and *COMT* genes and their gene-gene interactions with antipsychotic treatment response in patients with schizophrenia. A total of 101 patients with schizophrenia were recruited and stratified in treatment responder and treatment resistant groups based on the published criteria of resistant to treatment using PANSS. Clinical and demographic factors were analyzed. Genomic DNA was extracted from whole blood and genotyping for the four polymorphisms were done by ARMS-PCR, PCR-RFLP and gap-PCR. Gene-gene interactions were analyzed by logistic regression. In case of *DRD2* A-241G, G allele was significantly associated with resistant to treatment. Regarding *DRD4* 120-bp duplication, 240/240 genotype was significantly associated with resistant to treatment comparing to other genotypes in a dominant model. The genotype combination of *DRD4* 240/240 and *COMT* Val/Val was significantly associated with treatment resistant. Among *DRD2* AA genotype, *COMT* met allele carriers which also had a 120 bp allele of *DRD4* had a significantly better response to antipsychotics. Moreover, analysis of clinical and demographic factors demonstrated a significantly longer duration of hospitalization and higher chlorpromazine-equivalent daily dose in resistant to treatment patients. Discovering the polymorphisms which effect treatment response to antipsychotics will provide the possibility of genetic screening before starting an antipsychotic treatment which enhances the chance of responding to antipsychotics and decreases drugs side effects and costs.

## Background

Schizophrenia (SCZ) is a chronic, hereditary and disabling neuropsychiatric disorder with a worldwide prevalence of approximately 1% [1, 2]. In the etiology of SCZ, genetic factors are thought to play an important role, the heritability currently ranges from 64 to 81%; although, genetic mechanisms remain unclear [2–4]. The mainstay of schizophrenia treatment has been antipsychotic drugs over the past 60 years; however, clinical response differs significantly between patients, with an overall response rate of 50–70% [1, 5–7]. Many of patients with schizophrenia discontinue or switch drug regimens due to lack of treatment efficacy and/or drugs adverse side effects. In this view, there is a great need for the identification of predictive clinical and biological markers of treatment consequence [8, 9]. Pharmacogenetic biomarkers focus to predict which patients could improve with specified drugs according to genetic variants. Thus, genotype-based customized drug treatments may allow optimizing the antipsychotic treatment, while helping to minimize drugs side effects [10, 11].

To date, pharmacogenomics studies of response to treatment in schizophrenia, have typically focused on genes encoding for drug targets, called pharmacodynamics related genes. Many of the research investigating the association of pharmacodynamic genes with antipsychotic treatment response have concentrated on dopaminergic pathways, one of the primary mechanisms of function of antipsychotics, especially the gene coding for the dopamine D2 receptor(*DRD2* gene), which is a binding object for all available antipsychotic drugs. Dopaminergic gene SNPs are strongly related to drug sensitivity of antipsychotics; for example, several studies have indicated positive associations between *DRD2* gene and antipsychotic response [10–14].

Lingyue Ma and et al. by a systematic review and meta-analysis indicated that for Asian patients, at rs1799978(A241G) in *DRD2* gene AA genotype had a significantly greater improvement after risperidone therapy [15]. Three studies analyzed the association between rs1801028 in *DRD2* gene and antipsychotic or risperidone treatment in China and Slovenia; findings were inconsistent between the two studies [15–17].

Dopamine receptor D4(*DRD4*) is one of the main targets of antipsychotic drugs; furthermore, *DRD4* polymorphisms were associated with antipsychotic treatment response. A 120-bp duplication in the promoter region of *DRD4* is hypothesized to influence the clinical response to antipsychotic drugs [18–20].

Catechol-O-methyltransferase (COMT) is a major enzyme that inactivates dopamine in the prefrontal cortex by enzymatic degradation. The efficacy of antipsychotic drugs in patients with schizophrenia has been indicated to be related to rs4680 SNP in *COMT* gene; associated with a decreased therapeutic effect, leading to pharmacotherapy resistance. In addition, the *COMT* interacts with several genes to produce various phenotypes [20–24].

Based on our search in Pubmed and Google Scholar, this is the first study examining the frequencies of *DRD2* rs1799978, *DRD2* rs1801028 and *DRD4* 120-bp duplication in Iranian population. Although, the frequency of rs4680 in *COMT* gene is assessed in several studies with Iranian ethnicity. According to the mentioned studies, the overall frequency of G allele of rs4680 in a total sample size of n = 2200 is 51.57% in Iranian population [25–30].

Consequently, there is insufficient evidence of associations between polymorphisms in dopaminergic genes and antipsychotic treatment, particularly in Iran; thus, we carried out an association study between clinical factors and four polymorphisms in *DRD2*, *DRD4* and *COMT* genes, and antipsychotic treatment response in Iranian population. To best of our knowledge this is the first study in Iran that is investigating the effects of genetic factors and antipsychotic treatment response.

## Materials and methods

### Subjects

One hundred and one Iranian patients with schizophrenia were recruited to the study from Salamat psychiatric hospital of Khuzestan and Derakhshesh Rehabilitation center of Ahvaz. Inclusion criteria were as follows: 1) clinically confirmed diagnosis of schizophrenia according to the Diagnostic and Statistical Manual 5th Edition DSM-V criteria.2) hospitalized patients with schizophrenia whose drug intake were under supervision.3) at least 6 weeks under controlled antipsychotic treatment with adequate dose. exclusion criteria were as follows: 1) psychiatric comorbidities)such as mood disorders)2)other mental disorders 3)severe side effect of previous antipsychotic treatment 4)poor compliance to treatment5)substance abuse 6) substance-related disorder. The ethics committee of Ahvaz Jundishapur University of Medical Sciences approved this study (IR.AJUMS.MEDICINE.REC.1399.048). Informed consent was received from all patients/legal representatives enrolled in this study.

### Clinical and demographic information

Clinical and demographic information containing age, sex, marital status, education level, consumption of tobacco, start age of schizophrenia, duration of treatment and hospitalization, history of hospitalization and family history of psychiatric disorder were obtained. Furthermore, clinical symptoms before and after antipsychotic treatment, typical and atypical antipsychotics taken with daily doses were noted from patients medical files. The chlorpromazine-equivalent daily dose of antipsychotic drugs administered to patients was calculated based on published updated guidelines [31].

### Response and resistance to treatment

Response to treatment was evaluated by Persian version of PANSS- Positive and Negative Syndrome Scale- and psychiatrist diagnosis of remission in schizophrenia symptoms. The PANSS assessment was carried out by two trained and accurate psychologists after at least 6 weeks of under controlled antipsychotic treatment. PANSS is an internationally accepted scale used to measure symptoms severity in patients with schizophrenia and quantify clinical signs of schizophrenia. This scale contained 30 items separated into 3 scales; 7 items for the positive scale, 7 items for the negative scale and 16 items for the general psychopathological scale. The final score that indicates the severity of the disorder; is calculated by summing the answers for each question. Resistance to treatment was defined when the PANSS score was $\ge$4 in at least two of categories: P2 (conceptual disorganization), P3 (hallucinatory behavior), P6 (suspiciousness/persecution), G9 (unusual thought content) [17, 32].

### DNA sampling and genotyping

Genomic DNA was extracted from whole blood using the SinaClone DNA kit following the manufacturer instructions. Four polymorphisms in three candidate genes were analyzed in the present study: rs1799978 and rs1801028 in *DRD2*, rs4680 in *COMT* and 120-bp duplication polymorphism in *DRD4*.Genotyping of *DRD2* polymorphisms were conducted by using the amplification-refractory mutation system polymerase chain reaction (ARMS-PCR). In the case of rs1799978, two forward primers and one common reverse primer were as follow: F(allele A): 5’-CAGCCTGCAATCACAGCTTA-3’, F(allele G): 5’-CAGCCTGCAATCACAGCTTG-3 and R:5’-TGAAGCTGGACAGCTCTGC-3’. The two forward primers and one common reverse primer for rs1801028 were as follow: F (allele G): 5’-TGACTCTCCCCGACCCGTC-3’, F(allele C): 5’- TGACTCTCCCCGACCCGTG-3’ and R: 5’-GTTTGCCCATCTGTAAAGTGAGCAC-3’(primers used from Zalina Zahari et al [33]). The ARMS-PCRs were performed in two 15ul reactions for each patient, which contained an initial denaturation at 95 °C for 2 min followed by 35–36 cycles of denaturing at 95 °C for 30 s, annealing at 65 °C for 30s and extending at 72 °C for 45s. After that, a final extension at 72 °C for 5 min was applied.

*COMT* rs4680 was analyzed by polymerase chain reaction-restriction fragment length polymorphism (PCR- RFLP). The PCR was conducted in a 15ul reaction system which included an initial denaturation at 95 °C for 2 min followed by 35 cycles of denaturing at 95 °C for 30 s, annealing at 61 °C for 30 s and extending at 72 °C for 30s. After 35 cycles, it experienced a final extension at 72 °C for 5 min. NIaIII restriction enzyme was used for digestion; as explained by Qianqian He and et al [34]. Genotyping of 120-bp duplication polymorphism in *DRD4* was performed using the Gap-PCR (primers by [35]). The PCR was carried out in a15ul reaction system which contained a first denaturation at 95 °C for 2 min followed by 30 cycles of denaturing at 95 °C for 30 s, annealing at 66 °C for 30 s and extending at 72 °C for 55s. After 30 cycles, a final extension at 72 °C for 5 min was done.

### Statistical analysis

All data analysis was conducted using Statistical Package for the Social Sciences SPSS, Version 26. Continuous variables were expressed in the form of mean ± standard deviation. Kolmogorov–Smirnov test was applied to check whether the data were normally distributed; which normally distributed and abnormally distributed data between two groups were calculated respectively by t-test and Mann-Whitney U test. In order to assess the association between categorical variables of clinical parameters and genotype associations chi-square test was performed. Associations between polymorphisms and antipsychotic treatment response were analyzed under five genetic models including homozygous model, heterozygous model, recessive model, dominant model and co-dominant model. Logistic regression analysis was carried out in order to analyze these five genetic models. Adjusted odds ratios, adjusted P-values and 95% confident interval (95%CI) were calculated. Furthermore, logistic regression analysis was used to examine the effect of interaction among polymorphisms on antipsychotic treatment response. P < 0.05 was considered as statistical significance in all tests.

Gpower software version 3.1.9.7 was applied in order to analyze the power of logistic regression. According to Chen et al., 2010 [36] effect size in logistic regression can be classified as follows:

- Odds Ratio < 1.68 - Very small.
- 1.68 ≤ Odds Ratio < 3.47 - Small.
- 3.47 ≤ Odds Ratio < 6.71 - Medium.
- Odds Ratio ≥ 6.71 – Large.

The power analysis demonstrated that a sample size of 101 is sufficient to reveal a medium to large effect size with a minimum power of 80% at a significance level of 5%.

## Results

### Clinical and demographic characteristics of patients

One hundred and one patients with schizophrenia were included in this study (75 men and 26 women), which among them 51 patients were classified in the treatment-responder group and 50 patients met the criteria for treatment-resistance. There were significant differences between two groups in marital status, smoking, duration of hospitalization, chlorpromazine-equivalent daily dose and total PANSS score(Table 1). With regarding to the clinical characteristics, significantly longer duration of hospitalization, higher PANSS score and also higher chlorpromazine-equivalent daily dose were observed in treatment-resistant group(Table 1). Furthermore, married and smoker patients were significantly more in the treatment-responder group comparing to the treatment-resistant group.

**Table 1:** clinical and demographic characteristics of patients

Characteristics	Treatment-responder patients(n=51)	Treatment-resistant patients(n=50)	Statistics
Sex:			
Male	42(82.3%)	33(66%)	χ2 :3.532 P = 0.06
Female	9 (17.6%)	17(34%)	
Marital status:			
Married	15(31.9%)	6(13.3%)	χ2 = 4.505 P = 0.0337^*^
Unmarried	32(68.08%)	39(86.6%)	
Level of education:			
Illiterate	3(6.9%)	6(14.6%)	
Primary	23(53.4%)	24(58.5%)	
Secondary	14(32.5%)	9(21.9%)	χ2 = 2.2619 P = 0.519
university	3(6.9%)	2(4.8%)	
family history of psychiatric problem:			
1st degree	17(33.3%)	13(26%)	χ2 = 1.111 P = 0.29
2nd and 3rd degree	6(11.7%)s	9(18%)	
Smoking			
Yes	33(64.7%)	21(42%)	χ2 = 5.2317 P = 0.0221^*^
No	18(35.2%)	29(58%)	
Personal History of hospitalization(multiple relapse):			
Yes	28(54.9%)	36(72%)	χ2 = 3.179 P = 0.0745
No	23(45.09%)	14(28%)	
History of killing or harming others:			
Yes	17(33.3%)(3 killer)	12(24%)(2 killer)	χ2 = 1.0745 P = 0.299
No	34(66.6%)	38(76%)	
History of suicide or self-harm:			
Yes	9(17.6%)	6(12%)	χ2 = 0.636 P = 0.424
No	42(82.3%)	44(88%)	
Age (years)	41.8 ± 10.6	40 ± 9.36	t = 0.857 P = 0.394
Age of disease onset(years)	24.07 ± 10.89	20.78 ± 4.44	Z^‡^= -0.890 P = 0.373
Duration of hospitalization(months)^†^	6.84 ± 7.63	12.24 ± 9.57	Z^‡^= -4.318 P < 0.001^*^
Total PANSS^§^ score	65.27 ± 12.26	97.48 ± 18.60	t= -10.399 P < 0.001^*^
Chlorpromazine equivalent daily dose(mg)	731.73 ± 426.958	1034.66 ± 691.934	Z^‡^= -1.974 P = 0.048*

Notes: ^†^: Duration of hospitalization is related to the current episode. ^‡^: Mann-Whitney U test has been applied. ^§^: PANSS: positive and negative syndrome scale. ^*^: statistically significant result

The frequently prescribed antipsychotics were perphenazine(47%), olanzapine(37%), risperidone(32%), quetiapine(26%) and haloperidol (24%).

### Genetic analysis

#### Genotypic and allelic associations

The population was in Hardy-Weinberg equilibrium for the four polymorphisms genotyped in this study (p > 0.05). The G allele of *DRD2* A-241G was associated with increased risk of resistant to treatment when compared to A allele (OR(95%CI): 3.661,P = 0.02,Table 2). As for *DRD4* 120-bp duplication, there existed a considerable difference in allele distribution(P = 0.064,Table 2), indicating a higher frequency of 240-bp allele in treatment-resistant patients; although, the P value was not significant.

**Table 2:** Genotype and allele frequencies of polymorphisms within *DRD2*, *COMT* and *DRD4*

Gene polymorphism	Genotype frequencies			Allele frequencies	
*DRD2*/A-241G (rs1799978)	AA	AG	GG	A	G
Treatment-responder patients(n=51)	47(92.2%)	4(7.8%)	0	98(96.1%)	4 (3.9%)
Treatment-resistant patients(n=50)	39(78%)	9(18%)	2(4%)	87(87%)	13 (13%)
Statistics	P value*:0.065			P value*: 0.020 ** **OR(95%CI):3.6611(1.151–11.646)**	
*DRD2*/Ser311Cys (rs1801028)	GG	GC	CC	G	C
Treatment-responder patients(n = 51)	50(98%)	1(1.9%)	0	101(99%)	1(0.98%)
Treatment-resistant patients(n = 50)	48(96%)	2(4%)	0	98(98%)	2(2%)
Statistics	P value*=0.617			P value*: 0.619 OR(95%CI):2.061(0.184–23.099)	
*COMT*/Val158Met (rs4680)	GG	GA	AA	G	A
Treatment-responder patients(n = 51)	18(35.3%)	23(45.1%)	10(19.6%)	59(57.8%)	43(42.1%)
Treatment-resistant patients(n = 50)	24(48%)	18(36%)	8(16%)	66(66%)	34(34%)
Statistics	P value*=0.431			P value*:0.233 OR(95%CI):0.707(0.184–23.099)	
*DRD4* 120-bp duplication	240/240	240/120	120/120	240bp	120bp
Treatment-responder patients(n = 51)	8(15.7%)	33(64.7%)	10(19.6%)	49(48%)	53(51.9%)
Treatment-resistant patients(n = 50)	17(34%)	27(54%)	6(12%)	61(61%)	39(39%)
Statistics	P value*:0.089			P value*:0.064 OR(95%CI):0.591(0.338–1.034)	

*:P values were obtained using X^2^ tests. **:statistically significant result

Abbreviations: *COMT*: catechol-O-methyltransferase; *DRD2*:dopamine receptor D2; *DRD4*: dopamine receptor D4; OR: odds ratio

The logistic regression analysis revealed that regarding *DRD4* 120-bp duplication, patients with 120/120 and 240/120 had a lower risk of developing resistant to treatment as compared to patients with 240/240 genotype(AOR(95%CI): 0.196, P value: 0.033, Table 3). Moreover, regarding *DRD4* 120-bp duplication, homozygous and heterozygous genetic models indicated relations with antipsychotic treatment response, however it did not reach the significance level(P = 0.055 and P = 0.053 Respectively.

**Table 3:** Associations between *DRD2, COMT* and *DRD4* polymorphisms and antipsychotic treatment response under five genetic models by using chi-square and logistic regression for the AORs

Gene polymorphism (W>M)	Heterozygous model WW vs WM	Heterozygous model WW vs WM	Recessive model WW + WM vs MM	Dominant model WW vs WM + MM	Codominant model WW + MM vs WM
	P value OR(95%CI) A P value AOR^†^(95%CI)	P value OR(95%CI) A P value AOR^†^(95%CI)	P value OR(95%CI) A P value AOR^†^(95%CI)	P value OR(95%CI) A P value AOR^†^(95%CI)	P value OR(95%CI) A P value AOR^†^(95%CI)
*DRD2* A-241G rs1799978 (A > G)	0.214	0.108	0.243	0.061	0.128
	-	2.712(0.775–9.483)	-	3.314(0.978–11.232)	2.579(0.739–9.003)
	1.000	0.154	1.000	0.137	0.155
	-	4.113(0.588–28.780)	-	4.296(0.630-29.275)	4.115(0.586–28.87)
*COMT* rs4680 (G > A) (Val > Met)	0.366	0.228	0.636	0.195	0.352
	0.60(0.041–2.753)	0.587(0.246–1.399)	0.781(0.280–2.175)	0.591(0.266–1.313)	0.685(0.308–1.522)
	0.563	0.392	0.498	0.605	0.295
	1.7160(0.275-10.693)	0.552(0.142–2.147)	1.727(0.356–8.383)	0.725(0.215–2.446)	0.509(0.143–1.804)
*DRD4* 120bp duplication (240bp > 120 bp)	0.055	0.053	0.295	0.033*	0.273
	0.282(0.076–1.052)	0.385(0.144–1.028)	0.559(0.280–2.175)	**0.361(0.139–0.938)**	0.640(0.288–1.424)
	0.994	0.074	0.308	0.043*	0.244
	0.001>(0.001> -_)	0.243(0.052–1.148)	0.334(0.041–2.753)	**0.196(0.041–0.949)**	0.461(0.126–1.695)

Abbreviations: OR: odds ratio, AOR: adjusted odds ratio, *COMT*: catechol-O-methyltransferase; *DRD2*: dopamine receptor D2; *DRD4*: dopamine receptor D4, W: wild allele (more frequent allele), M: mutant allele(less frequent allele). A P value: adjusted P value. Notes: *: statistical significance, ^†^: AOR for sex, age, marital status, age of disease onset and duration of hospitalization

#### Gene-gene interaction analysis

Whether the presence of three polymorphism’s genotypes could influence the risk for treatment resistant to antipsychotic drugs was determined between *DRD4* 120-bp duplication, *COMT* rs4680 and *DRD2* A-241G. We carried out all possible subgroup analyses; which the significant interactions are indicated in Table 4. In the *COMT* Val/Val subset, we found significant association of the *DRD4* genotype with antipsychotics treatment response; where the combination of *COMT* Val/Val genotype and *DRD4* 240/240 genotype had a high risk for developing treatment- resistance(OR(95%CI) = 3.232(1.056–9.892), P = 0.04). Also, among patients with *COMT* Val/Met - Met/Met genotypes(Met allele carriers) those whose genotypes where AA for *DRD2* A-241G were significantly more likely to respond to antipsychotic drugs as compared to other genotype combinations(OR(95%CI) = 2.540(1.138–5.668), P = 0.023).

**Table 4:** Gene-gene Interaction analysis for 2 and 3 locus models by using logistic regression

*polymorphism1*	*polymorphism2*		*OR (95%CI)*	*P value*	*Genotype combination*	*Respond-ers* *(N = 51)* ^*†*^	*Resist-ants* *(N = 50)* ^*‡*^
*DRD4* 120-bp duplication 240/240	*COMT* rs4680 Val/Val		3.232(1.056–9.892)	**0.040**	240/240 and Val/Val	5	13
*DRD2* A-241G AA	*COMT* rs4680 Val/Met-Met/Met		2.540(1.138–5.668)	**0.023**	AA and Val/Met-Met/Met	30	18
*DRD2* A-241G AA	*DRD4* 120-bp duplication 120/240 − 120/120		3.000(1.279–7.035)	**0.012**	AA And 120/240 − 120/120	38	26
*polymorphism1*	*SNP2*	*SNP3*	*OR (95%CI)*	*P value*	*Genotype combination*	*Respond-ers* *(N = 51)* ^*a*^	*Resist-ants* *(N = 50)* ^*b*^
*DRD4* 120-bp duplication 120/240 − 120/120	*DRD2 A241G* *AA*	*COMT* rs4680 Val/Met-Met/Met	2.363(1.057–5.281)	**0.036**	*AA and 120/240 − 120/120 and Val/Met-Met/Met*	28	17

Notes: †: treatment responder group, ‡: treatment resistant group

Furthermore, analyzing the interactions of *DRD2* A-241G and *DRD4* 120-bp duplication polymorphisms, revealed a significant association between *DRD2* AA genotype and *DRD4* 120 bp allele carriers(*DRD4* 120/240 − 120/120), patients with this genotype combination had a significantly better respond to antipsychotics(OR(95%CI) = 3.000(1.279–7.035), P = 0.012).

Additionally, logistic regression analysis indicated a significant interaction among *DRD2* A-241G, *DRD4* 120-bp duplication and *COMT* rs4680 polymorphism’s; where patients with AA − 120/240 or 120/120 - Val/Met or Met/Met showed a significantly better respond to antipsychotics when comparing to patients with GA or GG-240/240-Val/Val genotype(OR(95%CI) = 2.363(1.057–5.281), P = 0.036).

## Discussion

The key findings of the present study were as follows. First, our genetic analysis for *DRD2* A-241G(rs1799978) polymorphism detected a significantly higher frequency of G allele in resistant to treatment patients in comparison with responders. A possible explanation for this association could be that since DRD2 binds to dopamine and is a G-protein coupled receptor, A-241G polymorphism is considered to be related to DRD2 density and affinity [37]. Furthermore, regarding DRD2, it is recorded that this receptor lonely could adjust effects of atypical antipsychotics; suggesting that DRD2 plays a substantial role in patients response to atypical antipsychotics [15]. In addition, Mingzhe Zhao et al. carried out a genome-wide and whole exome sequencing joint study analyzing both the common and rare genetic variants associated with risperidone treatment response in patients with schizophrenia. Significantly, after combining the findings of GWAS and WES they identified signaling pathways which were all associated with DRD2 and HTR2A, targets of risperidone [38]. Interestingly, consistent with our result, Escamilla R et al. in Mexico reported a significantly higher frequency of G allele in resistant to antipsychotic treatment patients [10]. Also, a meta-analysis reported a significant association between AA genotype and greater improvement after risperidone therapy in Asian patients [15]. However, inconsistently to our result, Yan P et al. studying 267 Han Chinese patients, documented a significantly superior response to olanzapine in G allele carriers comparing to wild AA patients with schizophrenia [37]. There are reasonable arguments to explain the disagreement observed in this study. First, diverse ethnicities as genetic background plays an important role in antipsychotic treatment response. Second, response phenotype is a complicated trait, differences observed in different studies may be related to heterogeneity of definitions or criteria of response phenotype [15, 39]. Third, different sample sizes may result in various outcomes.

Second, in the case of the Ser311Cys (rs1801028) polymorphism in *DRD2* gene, Cys allele displayed a very low frequency in Iranian population, therefore we did not found any type of association related to this polymorphism. Consistent to our result, Terzić T et al. did not observe any association between *DRD2* Ser311Cys polymorphism and antipsychotic treatment response in 138 Slovenian patients with schizophrenia [17]. Although, a study which was carried out in 690 Han Chinese patients, revealed a significant association between *DRD2* Ser311Cys polymorphism and PANSS total improvement rates [16]. Since the genetic background is completely different in Han Chinese ethnicity compared to Iranian ethnicity and the sample size of the last study was larger than our study, the differences observed in these two studies could be explained.

Our third result, regarding *DRD4* 120-bp duplication polymorphism, documented a significant association between 240/240 genotype with resistant to treatment comparing to 120/240 and 120/120 genotypes. To the best of our knowledge this is the first time that this association is reported. The 120-bp duplication region which is located in the upstream of the first codon, includes common sequences for various transcriptional factors and the longer allele decreases transcription of *DRD4* gene [20, 40]. Limited studies exist around the association of *DRD4* 120-bp duplication polymorphism with antipsychotic response; and a review study mentioned this polymorphism as a newly discovered marker of antipsychotic treatment response [41]. Rudi Hwang et al. reported a significant association between 120 bp allele and non-responder group in African Americans, although they did not observe this association in Caucasians [42]. Again the variety in ethnicities results in different outcomes.

Fourth, in case of *COMT* Val158Met (rs4680) polymorphism, we did not observe genotypic and allelic association with antipsychotic treatment response. Consistent to our result, Hajj A et al. studying 100 Lebanese patients, did not detect association between *COMT* Val158Met and antipsychotic treatment response in their whole sample. Although, they noted a gender-related difference for *COMT* SNP. In 27 men involved in their study, they reported that Met allele carriers were more prone to be resistant to treatment in comparison with men with Val/Val genotype [32].

Fifth, our results revealed three two-way and one three-way gene-gene interaction in *DRD2* A-241G, *COMT* rs4680 and *DRD4* 120-bp duplication polymorphisms with antipsychotic treatment response. In case of these four gene-gene interactions, *COMT* Val158Met polymorphism was involved in three of them. First, we observed that in the *COMT* Val/Val subset, patients with *DRD4* 240/240 genotype had a significantly higher risk for resistant to treatment. To our knowledge this is the first time that this interaction is reported. Second, in the *DRD2* A-241G AA homozygotes, *COMT* Met allele carriers demonstrated a significantly better response to antipsychotics. Third, our three way gene-gene interaction analysis indicated that *DRD4* 120-bp allele carriers and *COMT* Met allele carriers among *DRD2* AA homozygotes had a significantly superior response to antipsychotic treatment. Interestingly, Rajagopal VM et al. observed that patients with *COMT* Val/Met or Met/Met genotype who also had *DRD4* 120-bp allele revealed a significantly better response to clozapine [20]. Additionally, a meta-analysis reported a significantly improved response in Met-allele carrier Asian and Caucasian patients [39]. COMT is a catabolic enzyme with functional connection to dopaminergic transmission, the first target of most antipsychotics [11, 39]. Protein function and structure studies indicate that COMT Val-Met substitution causes a disruption in enzyme stability and decreases enzymatic activity [11, 39]. Importantly, tonic-phasic dopamine hypothesis supports our findings; according to this hypothesis, the lower activity form of COMT enzyme encoded by Met-allele may increase tonic dopamine and reduce phasic dopamine subcortically. Thus, this hypothesis suggest that the Met allele would predict a greater response to atypical antipsychotics, as they are considered to increase tonic dopamine transmission [7, 43]. Finally, the fourth gene-gene interaction found in this study was between *DRD2* A-241G and DR4 120-bp duplication polymorphisms, where the combination of *DRD2* AA homozygotes and 120-bp allele carriers was found to be associated with an improved response to antipsychotics. To the best of our knowledge this is the first study analyzing the three way gene-gene interaction between *DRD2* A-241G, *DRD4* 120-bp duplication and *COMT* Val158Met polymorphisms together. Also, the first to analyze the interaction between *DRD2* A-241G with *DRD4* 120-bp duplication, and *DRD2* A-241G with *COMT* Val158Met.

Sixth, regarding our clinical and demographic results, we demonstrated that marriage and smoking were significantly associated with better response to antipsychotics. Furthermore, duration of hospitalization, total PANSS score and chlorpromazine equivalent daily dose were significantly higher in resistant to treatment patients. Interestingly, Escamilla R et al. in Mexico reported significantly more weeks of disease evolution in ultra-resistant to treatment patients. Also, they observed the most prolonged durations of untreated psychosis and an earlier age of schizophrenia onset in ultra-resistant to treatment group. Besides, they observed more married patients in the responder group, however it did not reach the significance level [10]. Furthermore, consistent to our result, Aline Hajj et al. in Lebanon which studied 100 patients with schizophrenia, revealed a significantly higher chlorpromazine equivalent daily dose in resistant to treatment patients; either when the resistant to treatment was according to BRPS scale or when the PANSS scale was applied as the criteria of treatment resistant [32].Different ethnicities and sample sizes may be a reasonable explanation for diverse results obtained in these studies.

However, considerable work has been carried out in order to recognize valid biomarkers to predict antipsychotic treatment response in patients with schizophrenia, data acquired is still inadequate and with the exclusion of singular samples, no general guidelines regarding possible utilization of pharmacogenetics in antipsychotic consumption in daily clinical practice, exist. The exceptions are related to CYP2D6 and CYP3A4 enzymes. Clinical Pharmacogenetics Implementation Consortium and the Dutch Pharmacogenetics Working Group published guidelines recommending CYP2D6 genotyping for patients under treatment with risperidone; DPWG recommends a decreased dose of risperidone in patients with the poor metabolizer phenotype of CYP2D6 and altering treatment to another drug or titration of the dose in patients who are classified as CYP2D6 ultrarapid metabolizers. Again DPWG together with the FDA, in treatment with aripiprazole, recommends a reduced dose for patients who are classified as CYP2D6 poor metabolizers. Furthermore, in cases where aripiprazole and CYP3A4 inhibitors are taken simultaneously, the FDA suggests a decrease in aripiprazole dose, with no determination of the patient’s phenotype. Nevertheless, these guidelines are advantageous, they are in no way adequate, therefore, more and more emphasizing the need for further research in this field [44].

Our sample size was relatively small especially the subgroups in gene-gene interactions were small and a few genotypes had limited carriers(GG genotype of *DRD2* A-241G polymorphism and GC of *DRD2* rs1801028); also CC genotype of *DRD2* rs1801028 was not observed in our sample. Furthermore, no multiple testing correction was used for P-values in gene-gene interaction analyses and we should mention the type I error possibility as a limitation. Besides, the studied population was only from one single ethnicity. Consequently, further investigations with larger sample sizes and meta-analyses, from various ethnicities analyzing several polymorphisms involved in pathways related to antipsychotics actions are warranted; in order to move toward personalized medicine in schizophrenia.

## Conclusion

In summary, our results suggest that *COMT*, *DRD2* and *DRD4* genes together and *DRD2* and *DRD4* genes separately, may effect and predict antipsychotic treatment response in Iranian population. This kind of study may provide the possibility of genetic screening before starting a new antipsychotic trial, resulting in a better chance to achieve the most effective treatment for each patient in a shorter period of time, decreasing costs and minimizing adverse side effects of drugs.

## Data Availability

All data generated or analyzed during this study are included in this published article.
